# Correction: Dobrovskiy et al. The Transport Properties of Semi-Crystalline Polyetherimide BPDA-P3 in Amorphous and Ordered States: Computer Simulations. *Membranes* 2022, *12*, 856

**DOI:** 10.3390/membranes14050101

**Published:** 2024-04-29

**Authors:** Alexey Y. Dobrovskiy, Victor M. Nazarychev, Igor V. Volgin, Sergey V. Lyulin

**Affiliations:** Institute of Macromolecular Compounds, Russian Academy of Sciences, Bolshoj pr. 31 (V.O.), 199004 St. Petersburg, Russia

The authors wish to make a change to the published paper [1], as several mistakes have been made. 

In the original publication, there was a mistake in Figure 7 as published.

The calculation of the diffusion coefficients was carried out incorrectly due to a scaling error in the x-axis when processing the time dependence of the mean-squared displacement of gas molecules (Figures S4 and S5 in the Supporting Information). The corrected Figure 7 appears below:

**Figure 7:**
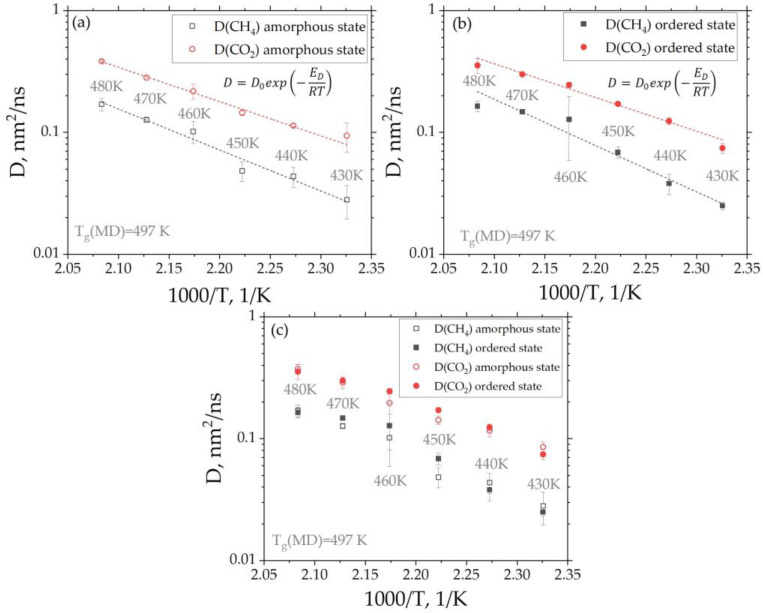
Dependence of the diffusion coefficients of the CH_4_ and CO_2_ gases in amorphous (**a**) and ordered (**b**) states in BPDA-P3 on the reciprocal temperature. Dashed fitting lines were obtained by approximation of the diffusion coefficient values using the Arrhenius law (Equation (4)). (**c**) Dependence of the diffusion coefficients on the reciprocal temperature for both gases in amorphous and ordered states. The BPDA-P3 glass transition temperature shown in (**a**–**c**) was obtained in our previous study using the all-atom Molecular Dynamics simulations.

In the original publication, there was a mistake in Figure 9 as published. 

The recalculation of the diffusion coefficients led to a change in the values of the permeability in Figure 9. The corrected Figure 9 appears below. 

**Figure 9:**
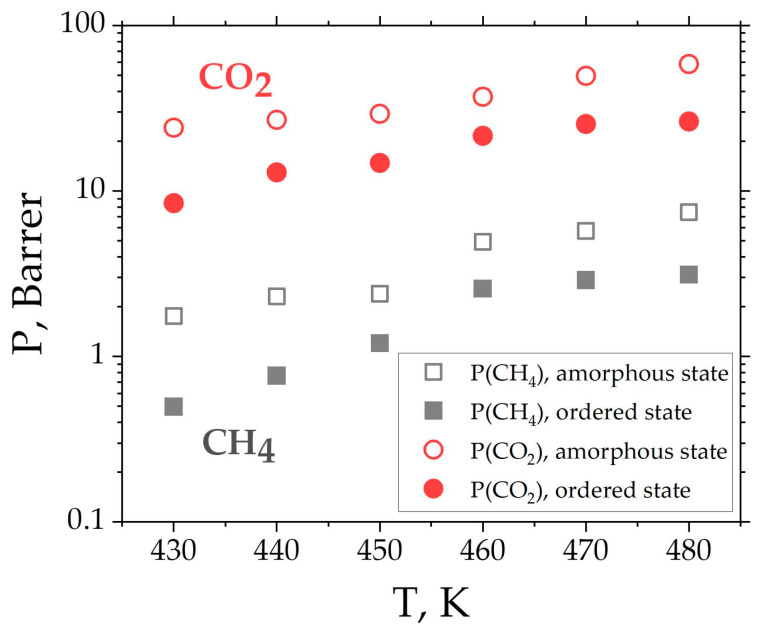
Temperature dependence of CH_4_ and CO_2_ permeability *P* of BPDA-P3 in amorphous and ordered states.

In the original publication, there was a mistake in Table 1.

Taking into account the recalculated diffusion coefficients, the activation energies in the table have to be updated. The correct Table 1 with activation energies is presented below. 

**Table 1:** Activation energy *E_a_* of diffusion of the CH_4_ and CO_2_ gases for BPDA-P3 in amorphous and ordered states.

	Amorphous State		Ordered State	
	CH_4_	CO_2_	CH_4_	CO_2_
*E_a_*, kJ/mol	64.4 ± 5.4	53.8 ± 1.1	72.6 ± 4.2	53.0 ± 3.6

There was an error in the original publication. Due to the calculation error, the conclusions applicable to the activation energy are incorrect. 

The following correction has been made to Results and Discussion, Transport Properties, paragraph 4. The Corrected parts are highlighted with bold font:

Comparing the resulting activation energies, we can conclude that in the ordered state, the diffusion activation energy is **only slightly higher (or remains unchanged taking into account the calculation error) than in the amorphous one**. In addition, *E_a_* for CO_2_ diffusion is lower in both states than *E_a_* for CH_4_, which is consistent with the values of the diffusion coefficient for these gases.

There was an error in the original publication. The article also contains a misprint regarding the BPDA-P3 glass transition temperature (*T_g_*).

A correction has been made to the abstract. The Corrected parts are highlighted with bold font:

The effect of polymer chain ordering on the transport properties of the polymer membrane was examined for the semi-crystalline heterocyclic polyetherimide (PEI) BPDA-P3 based on 3,3′,4,4′-biphenyltetracarboxylic dianhydride (BPDA) and diamine 1,4-bis [4-(4-aminophenoxy)phenoxy]benzene (P3). All-atom Molecular Dynamics (MD) simulations were used to investigate the gas diffusion process carried out through the pores of a free volume several nanometers in size. The long-term (~30 μs) MD simulations of BPDA-P3 were performed at T = 600 K, close to the experimental value of the melting temperature (Tm ≈ 577 K). It was found during the simulations that the transition of the PEI from an amorphous state to an ordered one occurred. We determined a decrease in solubility for both gases examined (CO_2_ and CH_4_), caused by the redistribution of free volume elements occurring during the structural ordering of the polymer chains in the glassy state (**Tg ≈ 487 K**). By analyzing the diffusion coefficients in the ordered state, the presence of gas diffusion anisotropy was found. However, the averaged values of the diffusion coefficients did not differ from each other in the amorphous and ordered states. Thus, permeability in the observed system is primarily determined by gas solubility, rather than by gas diffusion.

The following correction has been made to Results and Discussion, Transport Properties, paragraph 3:

The diffusion coefficients for the gases at temperatures below **Tg (≈487 K)** in the range of 430 up to 480 K were calculated using the corresponding MSD curves (see Figures S4 and S5 in Supporting Information). Below 430 K, it is necessary to significantly increase the duration of the simulations in order to achieve a normal diffusion regime. The resulting temperature dependence of the diffusion coefficients D for both gases corresponds to the Arrhenius law (Equation (4)) (see Figure 7a,b). The latter fact indicates that the diffusion activation energy at the temperatures considered is preserved, and hence, the diffusion mechanism does not change. The diffusion activation energies for the CH_4_ and CO_2_ gases in amorphous and ordered states are presented in Table 1.

The authors state that the scientific conclusions of the original article are unaffected. These corrections were approved by the Academic Editor. The original publication has also been updated.
