# Supplementary material for: The Transport Properties of Semi-Crystalline Polyetherimide BPDA-P3 in Amorphous and Ordered States: Computer Simulations
Source: Membranes (Basel). 2022 Aug 31;12(9):856. doi: 10.3390/membranes12090856 (PMC9504751; doi:10.3390/membranes12090856)
Supplement: Supplementary file 1 [file membranes-12-00856-s001.zip › membranes-1869600-supplementary.pdf]

membranes

Supporting Information

The Transport Properties of Semi-Crystalline Polyetherimide BPDA-P3 in Amorphous and Ordered States: Computer Simulations

Alexey Y. Dobrovskiy, Victor M. Nazarychev, Igor V. Volgin and Sergey V. Lyulin *

Institute of Macromolecular Compounds, Russian Academy of Sciences, Bolshoj pr. 31 (V.O.), St. Petersburg 199004, Russia
* Correspondence: s.v.lyulin@gmail.com; Tel.: +7-812-323-0216

We carried out additional calculations of the order parameter S_{or} so as to estimate its limiting values corresponding to the amorphous and ordered states. First, several sets of 100,000 randomly oriented unit vectors were generated in a spherical coordinate system ($\boldsymbol{r}$, θ, φ) in a sphere with unit radius $\boldsymbol{r}$. The azimuth angle φ of vectors in all sets has values from 0° to 360°, and the range of the polar angle θ varies for each set and has values from 90° to (90-$d\theta$)°, while $d\theta$ varies from 0° to 180°. Therefore, the vectors in a set are distributed in a cone with apex angle 2$d\theta$, where $d\theta$ corresponds to the angle between the z-axis and the generatrix of the cone. These sets allow us to simulate systems with polymer chains of various orientation directions. The amorphous state corresponds to $d\theta$ = 180°, while the ordered one corresponds to $d\theta$ ~ 0°, as shown in Figure S1a-d.

Then the S_{or} for each vector set was calculated and $S_{or}(d\theta)$ dependence was obtained (see Figure S1f). It can be seen from Figure S1 that small $d\theta$ = 10° (Figure S1a) or 30° (Figure S1b) vectors are oriented mainly along one direction (ordering director), leading to large values of the order parameter S_{or} close to 1. With an increase of $d\theta$ up to 90° (see Figure S1c), the vectors in the corresponding sets are less oriented relative to each other in one direction, which leads to a decrease in the values of the order parameter S_{or}, (see Figure S1f). With a further increase of $d\theta$ (from 90° to 180°), collinear vectors appear in the system: they lie on the same straight line, but are directed in different directions, see Figure S1d. Such collinear vectors contribute to the value of the order parameter S_{or}, which leads to the fact that its value does not reach 0. Such systems (with $d\theta$ ~ 180°) could correspond to the amorphous state of the polymer, where the polymer chains orient randomly, and values of S_{or} are in the range from 0 to 0.4. The ordered system corresponds to small $d\theta$ where S_{or} values are close to 1.

It should be noted that order parameter S_{or}, which is calculated regardless of the chosen axis, can take values from 0 to 1. In addition to S_{or}, the other frequently used order parameter $S(r)$, could be calculated through the Legendre polynomial as $^3/_2 \langle cos^2\theta(r)\rangle - {^1/_2}$, where $cos^2\theta(r)$ is the mean square of the angle cosine between vector associated with polymer chains and an axis along which ordering is considered. In the latter case $S(r)$ can take values from -0.5 to 1, but in current study this parameter was not calculated.

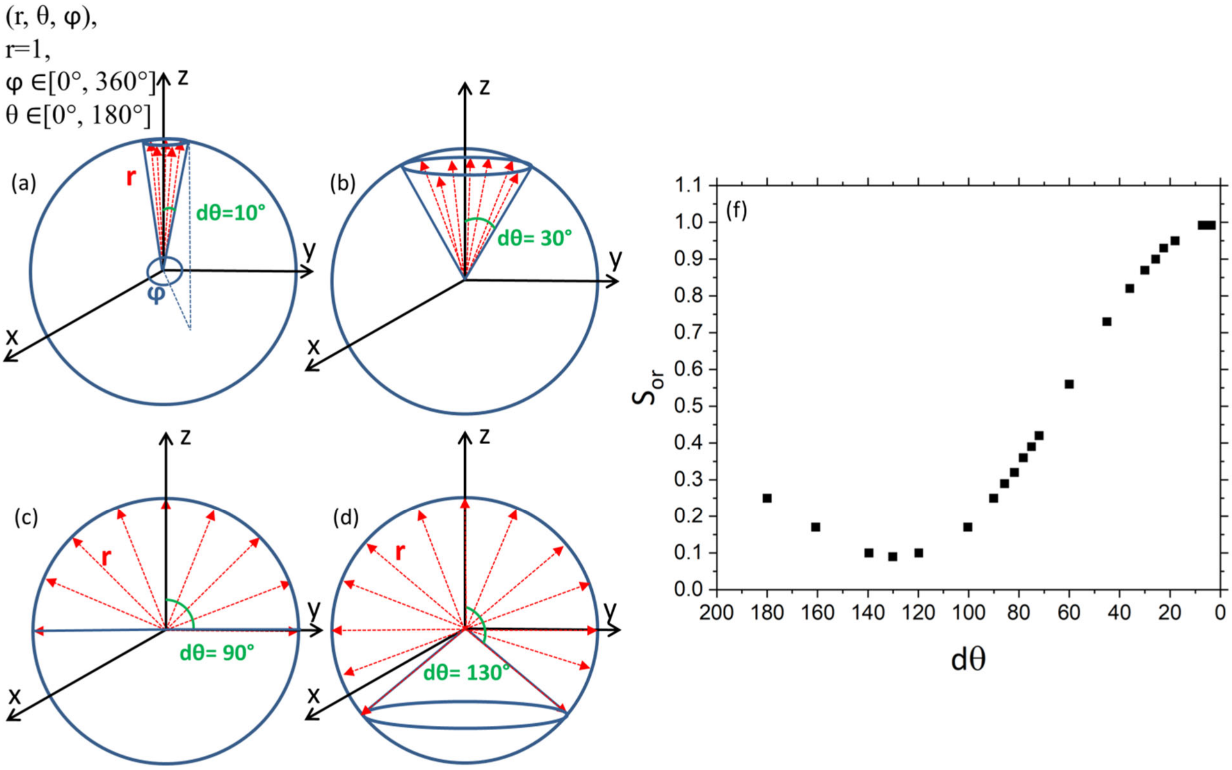

Figure S1. (a-d) Schematic representation of the method for calculating the order parameter S_{or} with different angle $d\theta$: 10°, 30°, 90° and 130°, respectively; (f) angular dependence of the order parameter S_{or}.

To study the structural properties of BPDA-P3, namely the change in the size of polymer chains over time, the mean values of the distance between the ends of the chains ($H_{e\text{-}e}$) and their radius of gyration (R_g) were calculated. R_g could be expressed as:

$$R_g = \left(\frac{\sum_i \|r_i\|^2 m_i}{\sum_i m_i}\right)^{\frac{1}{2}}, \quad \text{(S1)}$$

where m_i is the mass of i-th atom, r_i is the position of i-th atom relative to the center of mass of the molecule, and the brackets in || r_i || denote the modulus of the vector r_i.

Analysis of the time dependence of such geometric parameters as asphericity b and relative shape anisotropy κ^2 allows us to make a conclusion about the shape of polymer chains during the simulation time. The value of the asphericity parameter characterizes how much the shape of the polymer chains differs from the sphere. The relative anisotropy of the shape takes the value κ^2 =1 in the case of a completely unfolded chains. To calculate these characteristics, the main components of the inertia tensor of the polymer chains are used: $R_{g,x}$, $R_{g,y}$ and $R_{g,z}$:

$$b = R_{g,x}^2 - {}^1\!/_2\left(R_{g,z}^2 + R_{g,y}^2\right) \quad \text{(S2)}$$

$$\kappa^2 = \frac{b^2 + {}^3\!/_4\left(R_{g,y}^2 - R_{g,z}^2\right)^2}{R_g^4} \quad \text{(S3)}$$

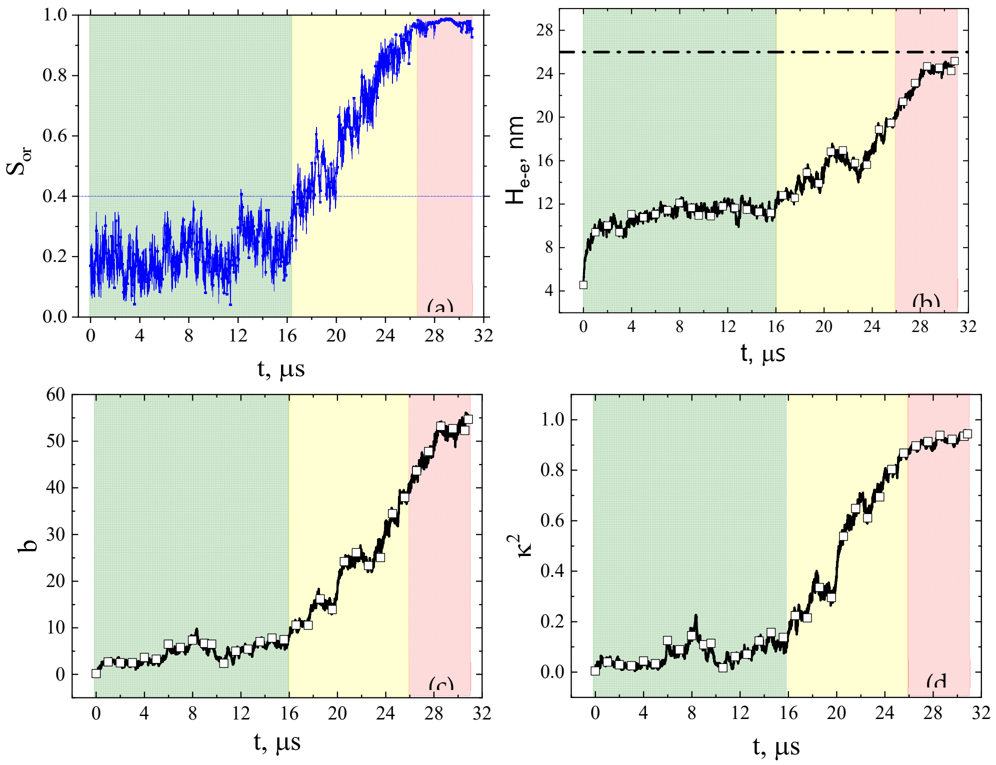

Figure S2. Time dependence of (a) order parameter S_{or}, (b) end-to-end distance H_{e-e}, (c) asphericity b, and (d) relative shape anisotropy κ^2 for BPDA-P3 at T=600 K.

From the analysis of the time dependence of H_{e-e} for BPDA-P3 chains shown in Figure S2b, it can be concluded that during the computer simulations, the chains gradually unfold from the coil state to their limit sizes (indicated by a dashed line). As shown in Figure S2c and Figure S2d, the polymer chains gradually unfold from an amorphous conformation, when the value of asphericity b and relative shape anisotropy κ^2 of the system is close to 0, gradually turning into an unfolded (ordered) state, when the time dependence of the parameters being studied reach a plateau.

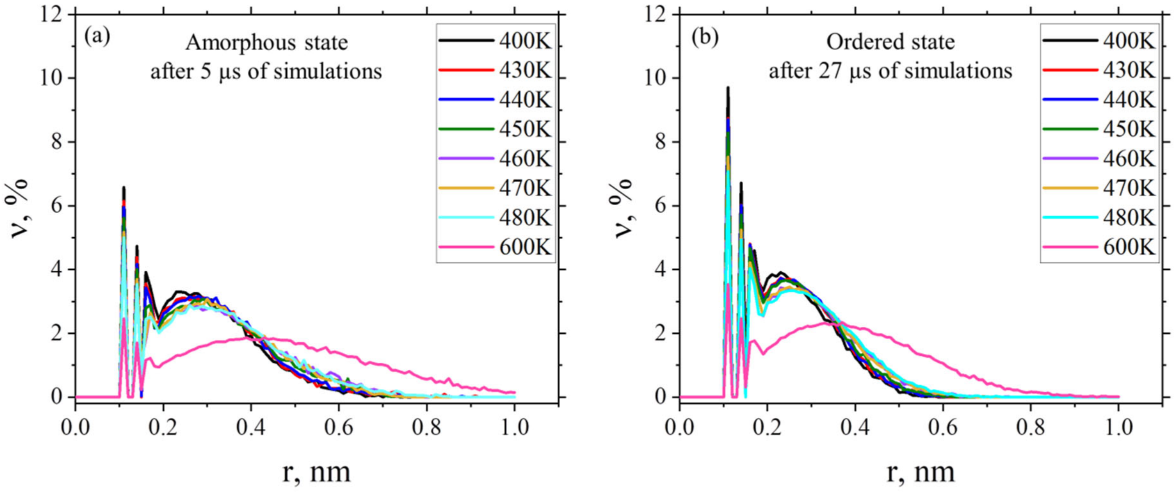

Figure S3. Free volume distribution (% of the total fraction of free volume) in amorphous (a) and ordered (b) states at different temperatures.

As follows from Figure S3 free volume distributions shift to the region of smaller values of free volume element`s sizes with temperature decrease, both in the amorphous and in the ordered state.

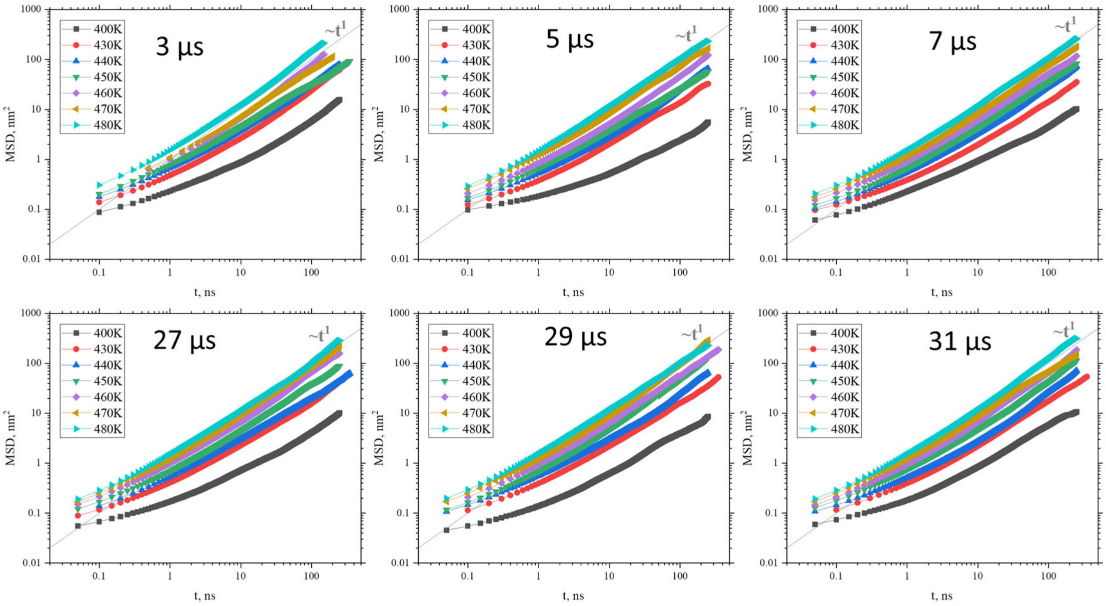

Figure S4. Time dependence of the mean-squared displacement for the CH_4 gas molecules in BPDA-P3 at different temperatures in the amorphous (for systems obtained after cooling after 3, 5, and 7 μs of simulations) and ordered (for systems obtained after cooling after 27, 29, and 31 μs of simulations) states.

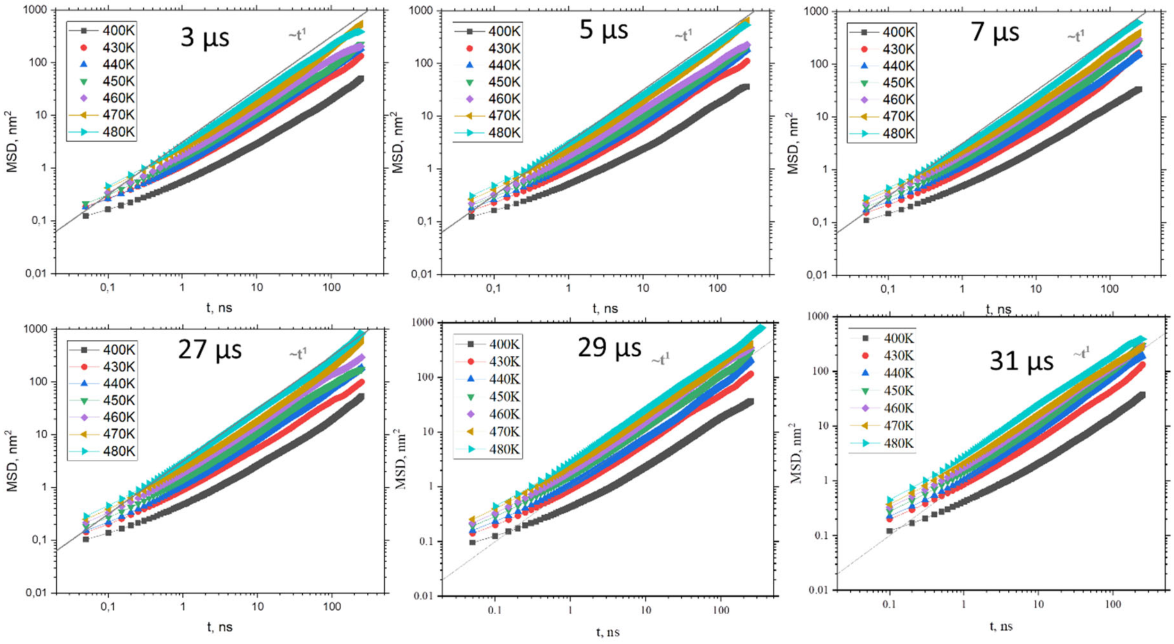

Figure S5. Time dependence of the mean-squared displacement for the CO_2 gas molecules in BPDA-P3 at different temperatures in the amorphous (for systems obtained after cooling after 3, 5, and 7 μs of simulations) and ordered (for systems obtained after cooling after 27, 29, and 31 μs of simulations) states.

The diffusion coefficients in the amorphous and ordered states were calculated from the mean-squared displacement (MSD) curves for CO_2 и CH_4 gas molecules. MSD of gas molecules was calculated from trajectories with 500 ns duration. The initial configurations for the trajectories were created by BPDA-P3 cooling from 600 K to the considered temperatures in the range from 480 to 430 K after 3, 5, 7, 27, 29 and 31 μs of simulations. The presented graphs show the MSD curves along the trajectory from 0 to 250 ns and the dotted line indicates guides to the eye which represent the linear MSD dependence in the normal diffusive regime (slope=1). On the MSD curve in log-log coordinates, an area with slope=1 was selected. This section of curve was approximated by a linear function $y=kt+b$. The resulting b value was used to calculate the diffusion coefficient.

Since in logarithmic coordinates the equation $\langle \Delta r^2 \rangle = 6Dt$ can be rewritten as: $log_{10}\langle \Delta r^2 \rangle = log_{10}t + log_{10}6D$, addend $log_{10}6D$ will correspond to the value of the parameter b in the linear approximation. Then we get that $D = \frac{10^b}{6}$.
